# Smoking and hepatoblastoma: confounding by birth weight?

**DOI:** 10.1038/sj.bjc.6601143

**Published:** 2003-07-29

**Authors:** L G Spector, J A Ross

**Affiliations:** 1 Division of Pediatrics Epidemiology & Clinical Research, Department of Pediatrics, University of Minnesota Medical School, Minneapolis, USA

**Sir**,

The finding reported by Pang *et al* (2003) that parental smoking increases the risk of hepatoblastoma (HB) in offspring is interesting, but we would like to suggest an alternative mechanism than that of direct carcinogenesis by tobacco metabolites. Recent reports indicate that the proportions of low and very low birth weight (LBW and VLBW) children among Japanese (Tanimura *et al*, 1998) and US (Feusner and Plaschkes, 2002) HB cases is greater than the proportions in their respective general populations. Also, survival of LBW and VLBW infants has improved since the 1980s (Avery and First, 1994), and the rate of HB increased significantly over roughly the same time period (at least in the US; Ross and Gurney, 1998). Taken together, these data strongly suggest that LBW and especially VLBW is a risk factor for HB. Meanwhile, cigarette smoking is an established risk factor for LBW (Horta *et al*, 1997). We would be interested to know if the authors could control for birth weight in their analysis.
